# Variety-Specific Metabolomic Configurations Underlie Differences in Seed Color, Nutritional Quality, and Antioxidant Activity of Faba Bean (*Vicia faba* L.)

**DOI:** 10.3390/plants15182848

**Published:** 2026-09-17

**Authors:** Xueqian Niu, Chengda Lu, Chengcheng Pei, Di Sun, Xin Zhao, Yang Li

**Affiliations:** 1 College of Agronomy, Shanxi Agricultural University, Taiyuan 030031, China; luchengdadeyouxiang@126.com (C.L.); 2304609281@sxau.edu.cn (C.P.); sundi@sxau.edu.cn (D.S.); 2 Shanxi Institute of Organic Dryland Farming, Shanxi Agricultural University, Taiyuan 030031, China; zhaoxin@sxau.edu.cn

**Keywords:** *Vicia faba* L., variety comparison, nutritional quality, antioxidant activity, untargeted metabolomics, functional components

## Abstract

Faba bean (*Vicia faba* L.) is a legume crop with diverse nutritional and bioactive properties, yet the metabolic basis underlying inter-varietal quality differences remains poorly understood. This study investigated four faba bean varieties (JD, GB, DB, and XJ) to test the hypothesis that variety-specific metabolomic configurations systematically govern phenotypic variations in seed color, nutritional composition, and antioxidant activity. Seed color parameters, proximate components (protein, soluble sugar, starch, and total dietary fiber), and antioxidant activities (DPPH and FRAP) were evaluated. Untargeted UPLC-MS/MS-based metabolomics was employed to characterize global metabolic profiles, screen differential metabolites, and perform KEGG pathway enrichment analysis, followed by TCMSP-based identification of bioactive compounds. JD exhibited the darkest seed color and the strongest antioxidant activity, whereas XJ showed the highest crude protein and total dietary fiber contents. Metabolomic profiling revealed that phenolic acids and flavonoids were predominantly up-regulated in JD, with coordinated down-regulation of multiple lipid species. KEGG enrichment indicated that JD’s antioxidant superiority was associated with activation of the flavonoid biosynthesis pathway and suppression of carotenoid biosynthesis. Ten JD-specific bioactive compounds were identified through TCMSP screening, among which gallic acid and licochalcone B exhibited strong DPPH radical scavenging and lipid peroxidation inhibitory activities, providing a material basis for the superior antioxidant capacity of JD. These findings demonstrate that variety-specific metabolomic configurations serve as the intrinsic basis for quality differentiation in faba bean and provide metabolite-level markers for breeding programs targeting enhanced antioxidant capacity and functional quality.

## 1. Introduction

Faba bean (*Vicia faba* L.) is a widely cultivated cool-season legume crop renowned for its high-quality plant protein, dietary fiber, and diverse bioactive compounds, making it a valuable resource for plant-based nutrition and functional food development [1,2]. It contributes significantly to food security and dietary improvement, while advances in processing technologies and protein functionality continue to enhance its value-added utilization [3,4,5]. In addition to basic macronutrients such as crude protein, starch, and soluble sugars, faba bean seeds are rich in secondary metabolites, including phenolic acids, flavonoids, terpenoids, and alkaloids, which exhibit a range of physiological activities, such as antioxidant, anti-inflammatory, and hypoglycemic effects. Among these, phenolic acids and flavonoids have garnered particular interest in recent years due to their potent bioactivities [6,7,8].

Different faba bean varieties display considerable natural variation in quality traits, including seed color, nutritional composition, and antioxidant activity, which provides a valuable germplasm resource for screening specialty varieties and supporting quality improvement [9,10]. Seed color serves not only as a key commercial trait but also as a reliable indicator of phenolic accumulation. Dark-colored seed coats consistently contain higher levels of total phenolics, flavonoids, proanthocyanidins, and anthocyanins than their light-colored counterparts, and correspondingly exhibit stronger in vitro antioxidant activity, suggesting that seed color could be used as a visual proxy for phenolic content [11,12]. At the molecular level, this phenotypic association is underpinned by transcriptional regulation of tannin and anthocyanin biosynthesis in the seed coat, with transcription factors such as TTG1 and TT8 playing critical roles [13,14]. In terms of nutritional composition, crude protein content varies from 20% to 34% across varieties, with marked differences in soluble sugars and total dietary fiber, whereas starch content appears to be less influenced by varietal background [15,16,17]. In addition to these nutritional components, faba bean seeds also contain anti-nutritional factors such as tannins, phytic acid, vicine, and convicine [18]. Tannins are mainly located in the seed coat, and their content is closely associated with seed coat color—dark-colored seed coats generally contain higher levels of tannins. These anti-nutritional factors should be considered in variety evaluation and germplasm utilization. Furthermore, post-harvest storage conditions—including temperature, humidity, and light exposure—can affect phenolic stability, thereby altering seed color and bioactivity and adding further complexity to quality evaluation [19]. Despite these findings, most current studies on faba bean quality remain confined to single nutritional indices or measurements of total phenolics and flavonoids. Systematic analyses of the compositional diversity of bioactive components, core functional substances, and their associated metabolic regulatory mechanisms are still lacking, which hampers the elucidation of the chemical basis of quality trait formation at the molecular level. Untargeted metabolomics enables simultaneous detection of differential metabolites across multiple chemical classes without pre-defined targets, offering an ideal approach for systematically dissecting inter-varietal metabolic differences and identifying core functional substances.

Untargeted metabolomics based on ultra-high-performance liquid chromatography-tandem mass spectrometry (UPLC-MS/MS) enables high-throughput qualitative and quantitative analysis of thousands of metabolites and has become a core tool for quality research in legume crops [20,21]. In recent years, this approach has been progressively applied to faba bean quality studies, where it has effectively distinguished metabolic profiles among varieties, screened differential metabolites associated with nutrition, flavor, and anti-nutritional factors, and elucidated regulatory networks via KEGG pathway enrichment analysis [22,23]. Its effectiveness in discovering bioactive metabolites has also been demonstrated in other edible legumes [24]. The metabolomic composition of faba bean is influenced by multiple factors, including genetic background, growing environment, and developmental stage, with flavonoids and phenolic acids representing the main sources of varietal differences [25,26]. As the largest class of plant secondary metabolites, the flavonoid biosynthetic network has been systematically characterized in model species; however, the accumulation patterns and regulatory pathways in different faba bean varieties remain poorly understood [27]. The Traditional Chinese Medicine Systems Pharmacology Database (TCMSP) integrates ADME properties—such as oral bioavailability and drug-likeness—of natural compounds, enabling efficient screening of functional components from large-scale metabolomic datasets [28]. These parameters, originally defined in a pharmaceutical context, are also applicable to prioritizing phenolic acids and flavonoids with favorable absorption properties and potential health benefits in faba bean. This combined strategy of untargeted metabolomics and TCMSP screening has been validated for bioactive compound identification in various edible legumes [29]. Nevertheless, current metabolomic investigations on faba bean have largely centered on low-tannin trait improvement and flavor compound identification, whereas integrated studies that link nutritional quality, antioxidant activity, and metabolomic profiles remain scarce, hampering a comprehensive understanding of the material basis and regulatory networks underlying quality trait formation.

To address these gaps, we hypothesized that inter-varietal quality differences in faba bean are not merely discrete variations in individual traits but are systematically governed by variety-specific metabolomic signatures. To test this hypothesis, four faba bean varieties were selected and comprehensively evaluated for seed color, nutritional composition, and antioxidant activities. Untargeted metabolomics was further employed to characterize inter-varietal metabolic differences, screen differential metabolites, perform KEGG pathway enrichment analysis, and identify core bioactive compounds via the TCMSP database. The overarching goal of this study was to elucidate the metabolomic basis of quality variation, clarify the functional distinctions among varieties, and provide a theoretical foundation for both specialty variety selection and value-added utilization of faba bean.

## 2. Materials and Methods

### 2.1. Materials

Four faba bean (*Vicia faba* L.) varieties were included in this study: JD (Jindou, a newly bred cultivar), GB (Gaobai, a landrace), DB (Dabaipi, a landrace), and XJ (Xinjing, a traditional cultivar). Detailed information on geographic origins, seed coat color, variety background, and maturity of the four varieties is provided in Supplementary Table S1. All varieties were sown in June 2025 at the Dongyang Experimental Station in Jinzhong City, Shanxi Province, China (37.55° N, 112.67° E; altitude 799 m), following a randomized complete block design with three replications. Plants were harvested at full maturity, and the seeds were threshed and naturally air-dried. For each variety, samples from three plots were pooled, pulverized in a ball mill, sieved through a 60-mesh screen, and stored at −80 °C until further analysis. The ground samples were divided into two aliquots: one for determination of color, nutritional components, and antioxidant activities, and the other for untargeted metabolomics analysis.

### 2.2. Determination of Quality Indicators

#### 2.2.1. Color Measurement

Ground faba bean powder was placed into a standard sample dish, and color parameters were measured using a non-contact color difference meter (Xrite VS450, X-Rite Inc., Grand Rapids, MI, USA). The L* (lightness), a* (redness/greenness), and b* (yellowness/blueness) values were recorded, with higher L* indicating greater brightness, positive a* indicating redness, and positive b* indicating yellowness. Each sample was measured in triplicate, and results were expressed as mean ± standard deviation.

#### 2.2.2. Nutritional Composition Analysis

The contents of crude protein, soluble sugar, starch, and total dietary fiber (TDF) were determined following Chinese national standards GB 5009.5-2016, GB 5009.9-2016, GB 5009.88-2014, and GB/T 37493-2019, respectively [30,31,32,33]. Briefly, crude protein was determined by the Kjeldahl method using a nitrogen-to-protein conversion factor of 6.25. Soluble sugar was extracted with 80% ethanol, clarified by precipitation with zinc sulfate and potassium ferrocyanide, and quantified by the copper reduction–iodometric titration method. Starch content was measured after enzymatic hydrolysis with thermostable α-amylase and amyloglucosidase, with the resulting glucose quantified via the glucose oxidase method. TDF was analyzed using an enzymatic-gravimetric approach involving sequential digestion with thermostable α-amylase, protease, and amyloglucosidase, followed by ethanol precipitation and gravimetric determination after correction for residual protein and ash.

#### 2.2.3. Antioxidant Activity Assays

The faba bean extract was prepared by extracting 1.0 g of sample powder with 10 mL of 80% methanol at room temperature for 30 min with shaking, followed by centrifugation at 8000× *g* for 10 min. The supernatant was collected and used for antioxidant assays.

**DPPH Radical Scavenging Activity Assay**

The DPPH radical scavenging activity was determined according to the method of Brand-Williams et al. with slight modifications [34]. Briefly, 7.89 mg of 2,2-diphenyl-1-picrylhydrazyl (DPPH) was dissolved in methanol (98%) and made up to 100 mL to prepare a 0.2 mmol/L DPPH solution, which was then stored in the dark at room temperature for 1 h. An aliquot of 200 μL of faba bean extract was mixed with 1 mL of DPPH solution and 800 μL of Tris-HCl buffer (0.77 M, pH 7.4). The mixture was incubated at 37 °C in the dark for 30 min. Methanol was used as a sample blank instead of the DPPH solution, and ascorbic acid (vitamin C) was used as a positive control. The absorbance was measured at 517 nm, and the DPPH radical scavenging activity was calculated using the following formula:

DPPH scavenging rate (%) = [1 − (A_sample/A_control)] × 100%

where A_sample is the absorbance of the sample group and A_control is the absorbance of the control group (DPPH solution plus solvent). Each sample was analyzed in triplicate, and the results were expressed as percentages (%).

**FRAP (Ferric Reducing Antioxidant Power) Assay**

The FRAP assay was performed according to the method of Benzie and Strain with modifications [35,36]. The FRAP working solution was freshly prepared by mixing 300 mmol/L acetate buffer (pH 3.6), 10 mmol/L TPTZ solution (in 40 mmol/L HCl), and 20 mmol/L FeCl_3_·6H_2_O solution at a ratio of 10:1:1 (*v*/*v*/*v*), and pre-warmed at 37 °C. An aliquot of 20 μL of faba bean extract was mixed with 180 μL of FRAP working solution (diluted 1:10) and incubated at 37 °C for 30 min. The absorbance was then measured at 593 nm. A standard curve was prepared using FeSO_4_·7H_2_O (0–1000 μmol/L), and the results were expressed as μmol Fe^2+^ equivalents per gram of sample (μmol/g). Each sample was analyzed in triplicate.

#### 2.2.4. Untargeted Metabolomics Analysis

**Sample Preparation and Extraction**

Samples were freeze-dried (Scientz-100F) and ground (MM 400, Retsch; 30 Hz, 1.5 min). Aliquots of 30 mg powder were extracted with 1500 μL of pre-cooled (−20 °C) 70% methanol containing an internal standard (1:50, *v*/*w*). The mixture was vortexed six times (30 s each, at 30 min intervals) and then centrifuged at 12,000 rpm for 3 min. The supernatant was collected and filtered through a 0.22 μm membrane prior to UPLC-MS/MS analysis.

**UPLC Conditions**

Separation was performed on a Waters ACQUITY UPLC HSS T3 column (1.8 μm, 2.1 mm × 100 mm) at 40 °C, with mobile phases of (A) 0.1% formic acid in water and (B) 0.1% formic acid in acetonitrile. The flow rate was 0.40 mL/min; injection volume was 4 μL. The gradient was 0–2 min, 95–25% A; 2–4 min, 25–1% A, held for 1.5 min; then returned to 95% A within 0.1 min and re-equilibrated for 1.4 min.

**MS Conditions**

Mass spectrometry was performed on an AB Sciex Triple TOF system with an ESI source, operated in IDA mode using Analyst TF 1.7.1. Source parameters: GAS1, 50 psi; GAS2, 60 psi; CUR, 35 psi; TEM, 550 °C; DP, ±80 V; ISVF, ±5500 V (positive) or −4500 V (negative). TOF MS scan: m/z 50–1250, accumulation time 200 ms. Product ion scan: m/z 50–1250, accumulation time 40 ms, collision energy ±30 V, intensity threshold 100 cps.

**Metabolite Identification Quality Control**

Equal volumes of all samples were pooled to prepare quality control (QC) samples, which were injected at regular intervals throughout the analytical sequence to monitor instrument stability. The TIC chromatograms of QC samples exhibited high overlay consistency, indicating good reproducibility of metabolite extraction and detection (Figure S1). The coefficient of variation (CV) of metabolites in QC samples was calculated; over 85% of metabolites had CV values below 0.5, demonstrating satisfactory data stability.

Metabolite identification confidence was evaluated using a three-tier classification system based on matching against the MWDB database:

Level 1 (confidently identified): MS/MS spectra and retention time matched with authentic standards (score ≥ 0.7);

Level 2 (putatively annotated): MS/MS spectra and retention time matched with database entries (score 0.5–0.7);

Level 3 (tentatively identified): identified based on accurate mass, retention time, and specific MRM ion pairs.

All metabolites reported in this study achieved Level 2 or higher annotation confidence.

**Identification of Key Bioactive Compounds**

Total phenolic and total flavonoid contents were estimated based on the relative peak intensities of phenolic acids and flavonoids identified through untargeted metabolomics. Our previous validation studies showed good correlations between the relative abundances of these compound classes and the corresponding values determined by the Folin–Ciocalteu and aluminum nitrate colorimetric methods, supporting the use of metabolomic relative quantification as an alternative indicator.

**Data Processing and Statistical Analysis**

Principal component analysis (PCA) was performed using the prcomp function in R (version 4.2.1) with log_2_ transformation and mean-centering. Hierarchical cluster analysis (HCA) and Pearson correlation heatmaps were generated using the R package ComplexHeatmap (version 2.16.0).

For two-group comparisons, differential metabolites were defined by variable importance in projection (VIP) > 1 and |log_2_ fold change (FC)| ≥ 1.0; for multi-group comparisons, by VIP > 1 and ANOVA *p* < 0.05. VIP values were extracted from orthogonal partial least squares discriminant analysis (OPLS-DA) models generated using the R package MetaboAnalystR (version 4.0), with log_2_ transformation and unit variance scaling. A 200-permutation test was performed to avoid overfitting.

**KEGG Annotation and Enrichment Analysis**

Identified metabolites were annotated using the KEGG Compound database (http://www.kegg.jp/kegg/compound/, accessed on 15 March 2026), and the annotated metabolites were subsequently mapped to the KEGG Pathway database (http://www.kegg.jp/kegg/pathway.html, accessed on 15 March 2026) for pathway enrichment analysis.

**Identification of Key Bioactive Compounds**

The TCMSP (Traditional Chinese Medicine Systems Pharmacology Database and Analysis Platform) is a dedicated database for systems pharmacology of traditional Chinese medicine, integrating chemical components, targets, and disease associations of herbal medicines. It allows screening of potential bioactive molecules based on drug-likeness (DL) and oral bioavailability (OB). According to the criteria of OB ≥ 5% and DL ≥ 0.14, the identified metabolites were recognized as key bioactive compounds [28]. OB ≥ 5% indicates that a compound possesses basic intestinal absorption capacity, while DL ≥ 0.14 requires structural similarity to known drug molecules. This threshold combination effectively enriches candidate compounds with both absorption potential and bioactivity. The related target and disease information for the identified metabolites was retrieved from the TCMSP database.

### 2.3. Statistical Analysis

All measurements for color, nutritional components, and antioxidant activities were performed in triplicate, and results were expressed as mean ± standard deviation (SD). Multivariate statistical analyses, including PCA and OPLS-DA, were performed using R software (version 4.3.0) with the ropls package (version 1.34.0) and factoextra package (version 1.0.7) for analysis and visualization. Hierarchical cluster analysis (HCA) was conducted using the pheatmap package (version 1.0.12). Pearson correlation heatmaps were generated using the corrplot package (version 0.92) and pheatmap package (version 1.0.12). Mean comparisons were performed using SPSS Statistics (version 26.0, IBM Corp., Armonk, NY, USA) with one-way analysis of variance (ANOVA) followed by Duncan’s multiple range test at a significance level of *p* < 0.05.

## 3. Results and Discussion

### 3.1. Differences in Color and Nutritional Quality Among Faba Bean Varieties

Seed color and core nutritional components of the four faba bean varieties were evaluated (Table 1). Considerable variations were observed among varieties in color parameters, major nutritional components, and total dietary fiber content. The coefficients of variation (CV) for crude protein and starch were both below 5%, indicating relatively low inter-varietal variability. In contrast, the color parameters a* and b* showed greater variation, with CV values exceeding 10%.

Regarding seed color, JD exhibited an L* value of 84.45, significantly lower than that of DB (the lightest-colored variety), while its a* and b* values were significantly higher than those of the other three varieties, indicating a darker seed color. Previous studies have shown that seed coat color in faba bean is closely associated with phenolic accumulation, with darker genotypes generally containing higher levels of total phenolics, flavonoids, and anthocyanins [37]. Consistently, the darker seed color of JD observed here suggests greater accumulation of phenolic secondary metabolites in its seeds.

In terms of nutritional composition, crude protein content among the tested varieties ranged from 28.86% to 31.78%, within the 20–35% range previously reported for faba bean protein [1]. Among the varieties, XJ exhibited the highest crude protein content (31.78%), significantly higher than that of GB and DB. Soluble sugar content was highest in JD and GB (approximately 10.4%), significantly exceeding that in XJ. These inter-varietal differences in soluble sugar content align with findings by Choi et al. [17] on the effects of dehulling on soluble sugar components in faba beans, suggesting that sugar composition may be largely influenced by varietal characteristics. Starch content across the four varieties ranged from 28.91% to 31.61%, with no significant differences among them. Total dietary fiber content was highest in XJ, significantly higher than in DB, while JD and GB showed intermediate levels. The total dietary fiber content of the four varieties ranged from 17.91% to 21.53%. This range is broadly consistent with the 9.18–25.47% reported across 68 faba bean accessions and the combined soluble and insoluble fiber range (approximately 11.3–17.1%) reported for 15 cultivars. The seed coat is the primary contributor to dietary fiber in faba bean, with values of 78.70–87.12 g/100 g reported in the testa of 20 Chinese cultivars [17]. Dehulling significantly reduces dietary fiber content, supporting the recommendation of whole-seed consumption for fiber-rich applications. The inter-varietal variation observed here further supports the potential of selecting varieties with enhanced fiber content.

### 3.2. Overall Metabolic Profiling of Different Faba Bean Varieties

**Figure 1:**
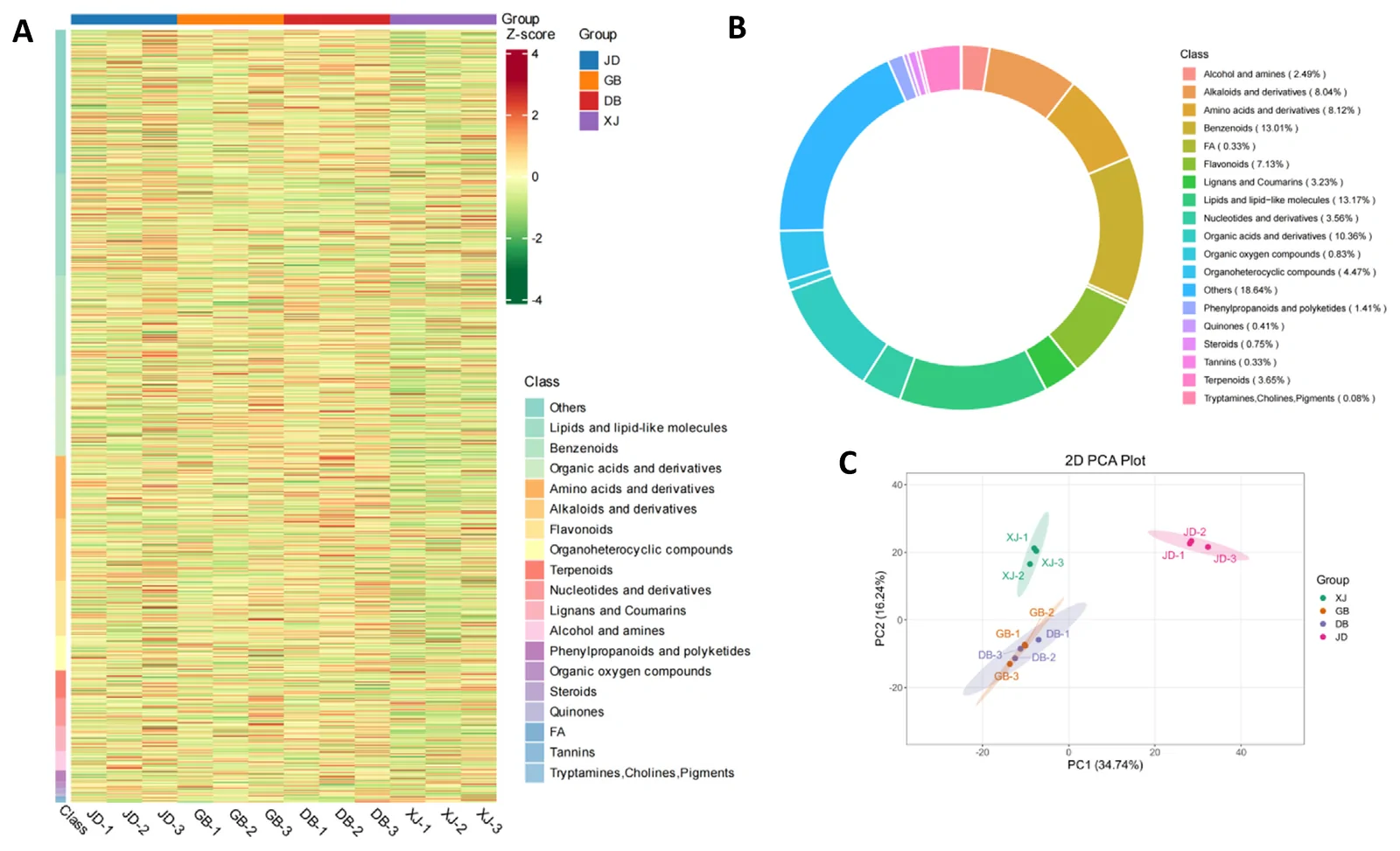
Metabolic profiling characteristics of faba bean. (**A**) Heatmap of all metabolites across different samples. (**B**) Classification of metabolites and the proportion of each category. (**C**) PCA score plot of different samples.

Untargeted metabolomics based on UPLC-MS/MS was used to profile the metabolome of the four faba bean varieties, identifying 2165 metabolites that were classified into 19 chemical categories (Figure 1A). Among primary metabolites, lipids and lipid-like molecules (13.17%) and organic acids and their derivatives (10.36%) accounted for the largest proportions, constituting the core metabolic backbone of faba bean (Figure 1B). Amino acids, nucleotides, and other basic metabolites were also detected, reflecting active primary metabolism in the seeds. Among secondary metabolites, benzenoids (13.01%) and flavonoids (7.13%) together comprised 20.14% of the total, representing the main source of phenolic bioactive components. Terpenoids, alkaloids, lignans, coumarins, and other secondary metabolites were also distributed across various categories, collectively contributing to the chemical diversity of the faba bean metabolome. In addition, 18.64% of metabolites were classified as “Others” because the number of identified compounds within their chemical categories was limited, primarily including ketones, saccharides, lactones, and vitamins. Such low-annotation categories may contain potential functional components associated with varietal differentiation that are underestimated due to insufficient database coverage or low mass spectrometry response. Future studies could employ multi-database searches, molecular networking-based annotation strategies, or targeted enrichment methods to further resolve the composition of these categories and their potential roles in faba bean quality formation.

Principal component analysis (PCA) showed that PC1 and PC2 explained 34.74% and 16.24% of the total variance, respectively, with a cumulative contribution of 50.98% (Figure 1C). Biological replicate samples of each variety clustered together on the score plot, indicating good experimental repeatability and that inter-variety metabolic differences outweighed intra-variety variations. Among the varieties, DB and GB were located close to each other, suggesting similar metabolic profiles. In contrast, XJ and JD were clearly separated from DB and GB, with JD showing the greatest separation along the positive direction of PC1, indicating the most distinctive metabolic composition among the four varieties. To further validate the reliability of variety discrimination, supervised PLS-DA was performed (Figure S2). The model showed clear separation among the four varieties with Q^2^ > 0.5, and the permutation test confirmed the model’s robustness (*p* < 0.05). These results indicate that the metabolic profiles of the four varieties are reliably distinguishable, supporting the subsequent analysis of differential metabolites.

The 2165 metabolites identified here are broadly consistent with the approximately 2500 metabolites previously reported in faba bean by Shi et al. [22]. In terms of class distribution, organic acids and their derivatives, along with lipids and lipid-like molecules, dominated the primary metabolite fraction. This aligns with the physiological role of faba bean seeds as energy reserves and storage tissues—organic acids participate in core energy metabolic pathways such as the tricarboxylic acid cycle, while lipids serve as important components of cell membrane structures [1,6]. Among secondary metabolites, benzenoids and flavonoids together accounted for 22.84% of the total, confirming faba bean as a typical phenolic-enriched crop [6] and supporting the view that phenolic compounds are important contributors to its antioxidant activity [7,8]. Metabolites that could not be clearly classified accounted for 20.9% of the total, a common occurrence in untargeted metabolomics due to the limited coverage of current databases [21]. The separation pattern in the PCA plot corresponded well with phenotypic differences in seed color and nutritional composition. JD showed the greatest separation along PC1 and also had the darkest seed color and strongest antioxidant activity. This suggests that metabolic specificity may underlie quality trait differentiation among varieties [10].

### 3.3. Analysis of Differential Metabolites Among Faba Bean Varieties

**Figure 2:**
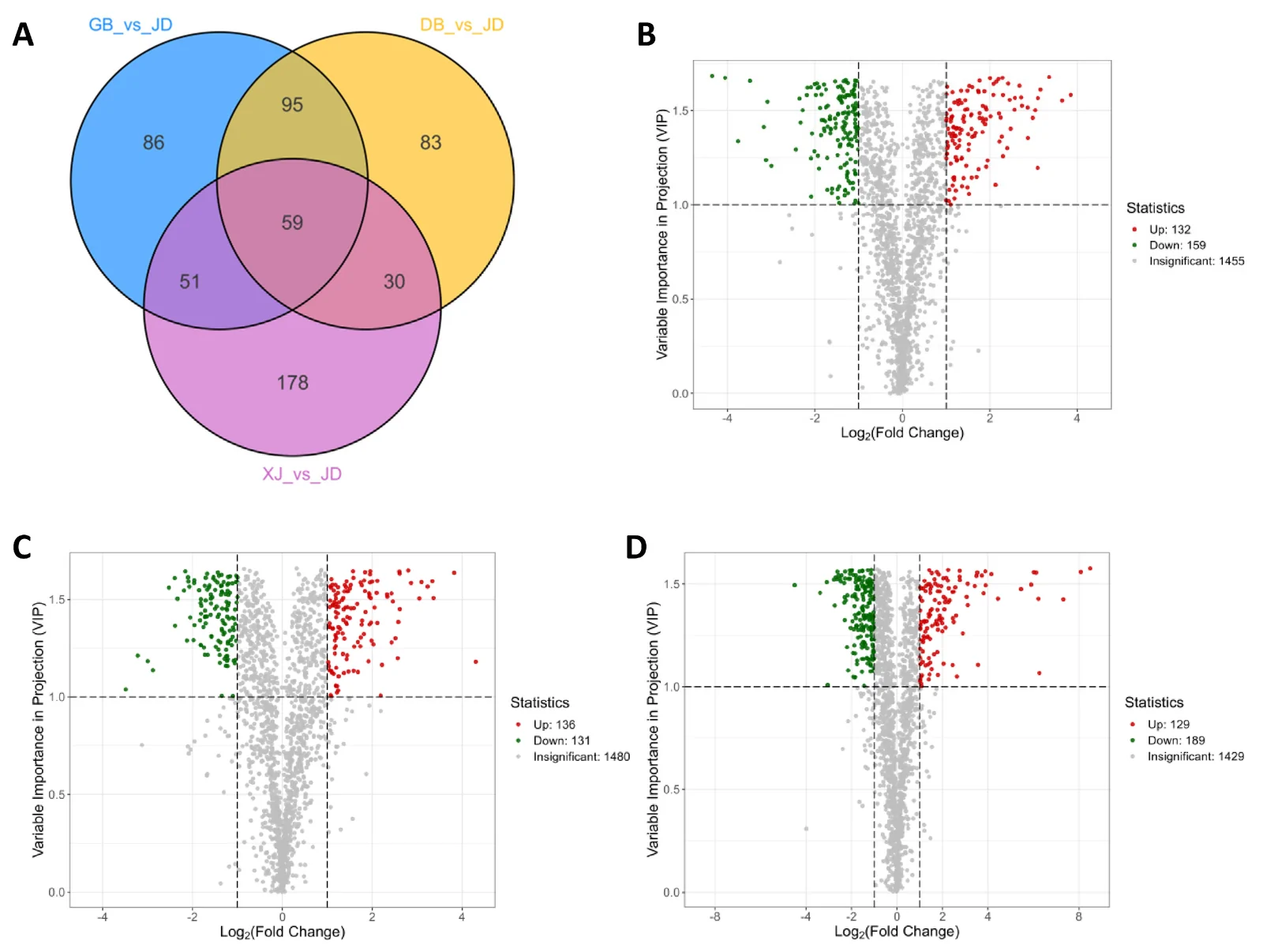
Venn diagram (**A**) and volcano plots (**B**–**D**) of differential metabolites between sample groups. Note: (**B**): GB vs. JD; (**C**): DB vs. JD; (**D**): XJ vs. JD.

Using VIP ≥ 1 and |log_2_FC| ≥ 1 as thresholds, we identified 291, 267, and 318 differential metabolites in the GB vs. JD, DB vs. JD, and XJ vs. JD comparisons, respectively, with JD as the reference (Figure 2A), indicating extensive metabolic differences between JD and the other varieties. Venn diagram analysis revealed 59 common differential metabolites across the three comparison groups, including 7 phenolic acids, 5 flavonoids, 6 organic acids and their derivatives, and 7 lipids and lipid-like molecules (detailed compound names are listed in Table S3), together comprising 42% of the shared differential metabolites. Analysis of up- and down-regulation patterns showed that phenolic acids and flavonoids in JD were predominantly up-regulated or unchanged across all three comparisons, whereas lipids and lipid-like molecules were mostly down-regulated.

Variety background is a core determinant of metabolomic composition in legumes, and genetic differences can drive significant metabolic divergence [22]. In the present study, a large number of differential metabolites (291–318) were detected between JD and GB, DB, and XJ (Figure 2B–D). The extent of these differences was consistent with the extensive metabolomic variation observed by Shi et al. [22] across different faba bean germplasms, further supporting the conclusion that genotype exerts a decisive influence on metabolic profiles in faba bean [10]. Among the 59 common differential metabolites shared by the three comparison groups, phenolic acids and flavonoids together accounted for more than 20%. Notably, phenolic acids outnumbered flavonoids (7 vs. 5), suggesting that phenolic acids may play a more prominent role than flavonoids in differentiating JD from the other varieties. The coordinated up-regulation of both classes points to a global activation of the phenylpropanoid pathway, rather than a branch-specific response. This pattern aligns with transcriptional regulation of phenolic metabolism, in which upstream regulators such as *MYB* and *bHLH* transcription factors coordinate the expression of multiple downstream genes [13,14]. The specific accumulation of these phenolic compounds—particularly phenolic acids and flavonoids—is thus a core metabolic feature distinguishing JD from the other varieties, consistent with its darker seed color phenotype [11]. Zanotto et al. [12] found that differences in phenolic composition among faba bean genotypes carrying different zero-tannin genes were mainly reflected in polymeric phenolics such as proanthocyanidins and anthocyanins, providing a genetic-level explanation for the up-regulation of phenolic acids and flavonoids in JD.

Meanwhile, lipids were generally down-regulated in JD, with more down-regulated than up-regulated compounds in all three comparison groups. Lipid synthesis and phenolic secondary metabolism share common precursors such as acetyl-CoA and phenylalanine, indicating a competitive relationship in carbon metabolic flux [6]. The widespread down-regulation of lipids in JD may reflect a greater allocation of carbon flux toward phenolic secondary metabolism rather than lipid biosynthesis. This concurrent up-regulation of phenolics and down-regulation of lipids suggests coordinated metabolic reprogramming at the carbon-partitioning level, supporting the view that variety-specific metabolomic configurations underlie quality differentiation in faba bean.

### 3.4. Analysis of JD-Specific Functional Compounds

**Figure 3:**
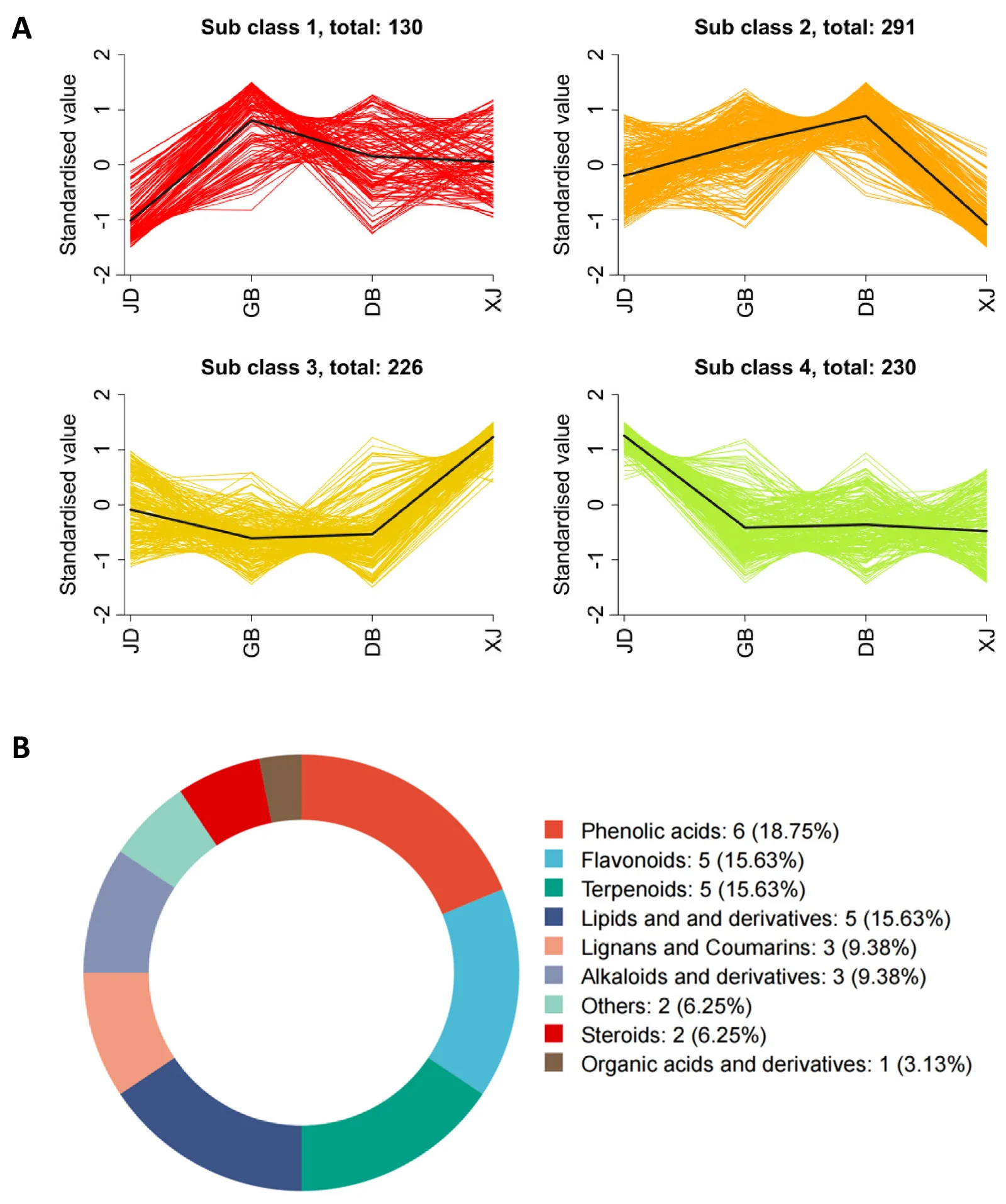
(**A**) Variation trends of metabolites across four varieties based on K-means clustering analysis. (**B**) Major functional active ingredients in JD classified by the TCMSP database.

The up-regulated metabolites identified in Section 3.3 represent candidate compounds that may contribute to JD’s functional advantages. However, not all differentially accumulated metabolites are bioactive. To prioritize those with potential functional significance, we performed TCMSP-based screening. Through K-means clustering analysis, the differential metabolites were divided into four metabolic clusters, of which Subclass 4—significantly enriched in JD—contained 230 differential metabolites (Figure 3A). Screening against the TCMSP database with oral bioavailability (OB) ≥ 5% and drug-likeness (DL) ≥ 0.14 as thresholds [28] identified 32 JD-specific candidate bioactive compounds, covering multiple categories including phenolic acids, flavonoids, terpenoids, and lipids (Figure 3B). Further selection yielded 10 representative functional metabolites (Table 2). Among these, the phenolic acids gallic acid, caffeic acid, and protocatechuic acid serve as core components underpinning its antioxidant activity; the flavonoid licochalcone B is directly associated with DPPH radical scavenging activity; and the specific accumulation of terpenoids such as soyasaponin I and ginsenoside Rh4, along with heptadecanoic acid, further expands the functional chemical repertoire of JD. The up-regulation of these compounds in JD is consistent with its higher DPPH radical scavenging activity, indicating that their coordinated accumulation underlies the antioxidant advantage of JD.

Gallic acid, caffeic acid, and protocatechuic acid have all been reported as the main phenolic acid components in faba bean seed coats [12,19]. Quantum chemical calculations have shown that the hydrogen-donating ability of phenolic hydroxyl groups and the ortho- and para-substituents on the benzene ring are key structural features determining the antioxidant activity of these three phenolic acids [38]. Gallic acid is a classic phenolic antioxidant, while caffeic acid and protocatechuic acid are significantly positively correlated with total phenolic content and FRAP values in faba bean [7,11]. Among the flavonoids, licochalcone B—a characteristic chalcone compound in legumes—has been confirmed to possess strong DPPH radical scavenging activity and the ability to inhibit lipid peroxidation [39,40], with 3 μg/mL sufficient to completely inhibit Fe(III)-ADP/NADPH-induced microsomal lipid peroxidation [41,42,43]. In addition, the up-regulation of soyasaponin I and ginsenoside Rh4, both triterpenoid saponins, in JD suggests that terpenoid metabolic pathways are also active in this variety [6]. Heptadecanoic acid, as the only significantly up-regulated characteristic lipid in JD, further contributes to the chemical diversity of JD’s metabolic advantage through its antioxidant activity.

Screening of the LC-MS/MS data for characteristic fragment ions led to the identification of two major anti-nutritional compounds in faba bean: vicine and convicine. Both compounds were detected in all four varieties, with variety-dependent differences in their relative contents (Table S2). JD exhibited the lowest vicine content, significantly lower than that of GB, DB, and XJ. For convicine, XJ showed the highest level, while GB and DB had the lowest, with JD showing an intermediate level. Vicine and convicine are hydrolyzed by β-glucosidase in the small intestine to their reactive aglycones, divicine and isouramil, which can trigger hemolytic anemia (favism) in individuals with glucose-6-phosphate dehydrogenase (G6PD) deficiency [44]. Therefore, the content of anti-nutritional factors is an important indicator for faba bean variety evaluation and germplasm utilization. The relatively low vicine content in JD, combined with its high antioxidant activity, suggests that this variety may have potential as a raw material for functional food development with reduced anti-nutritional risk.

### 3.5. Metabolic Pathway Enrichment Analysis of Differential Metabolites

**Figure 4:**
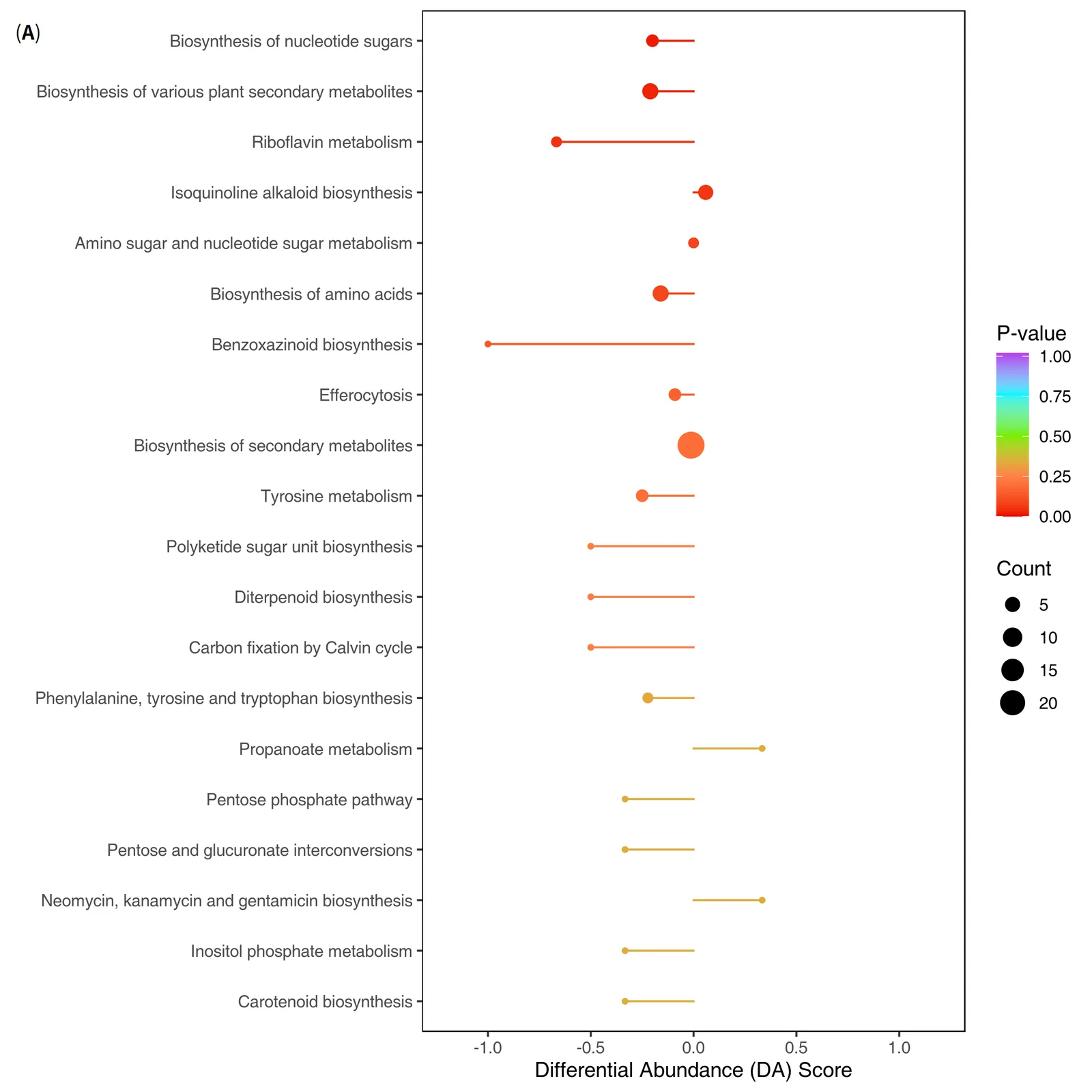

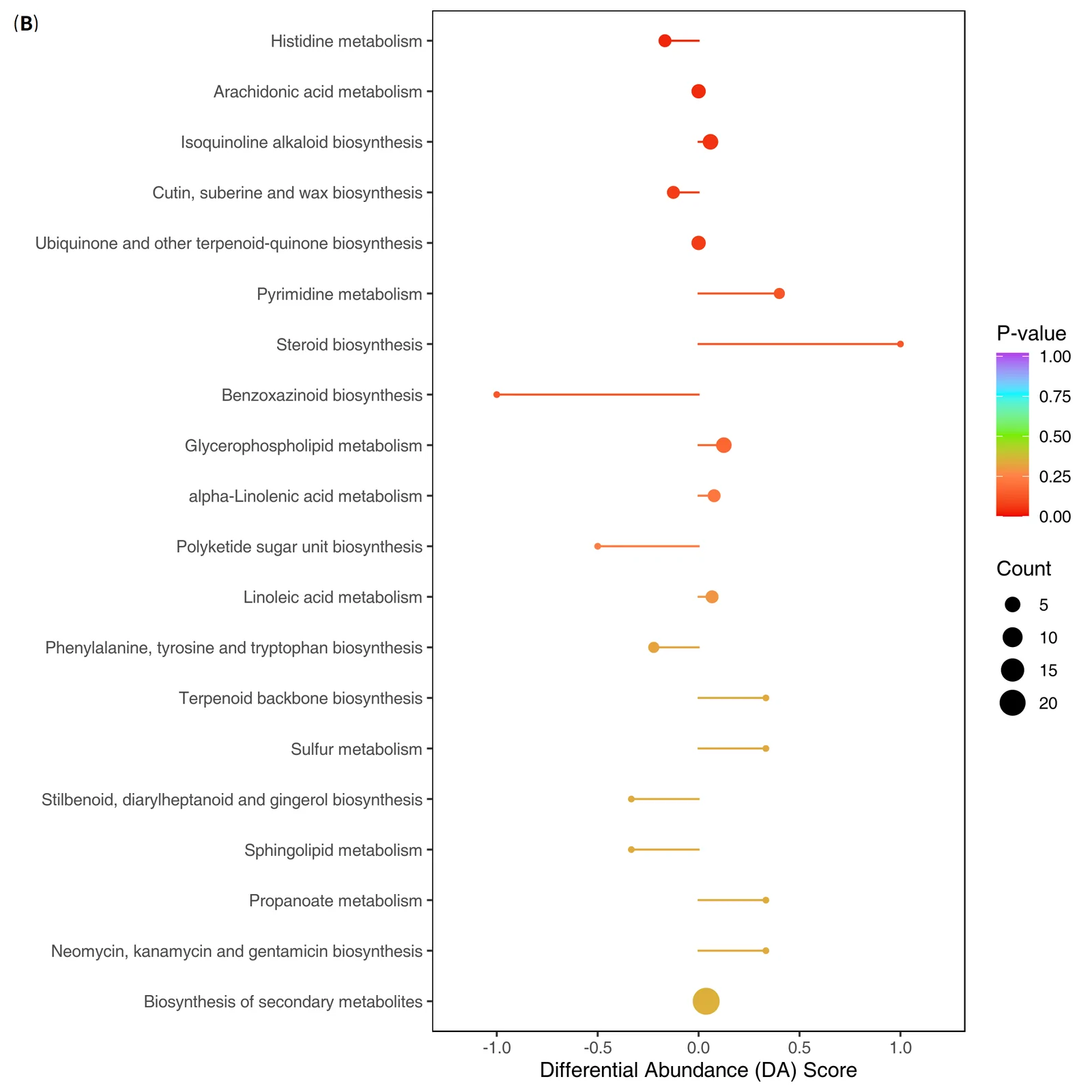

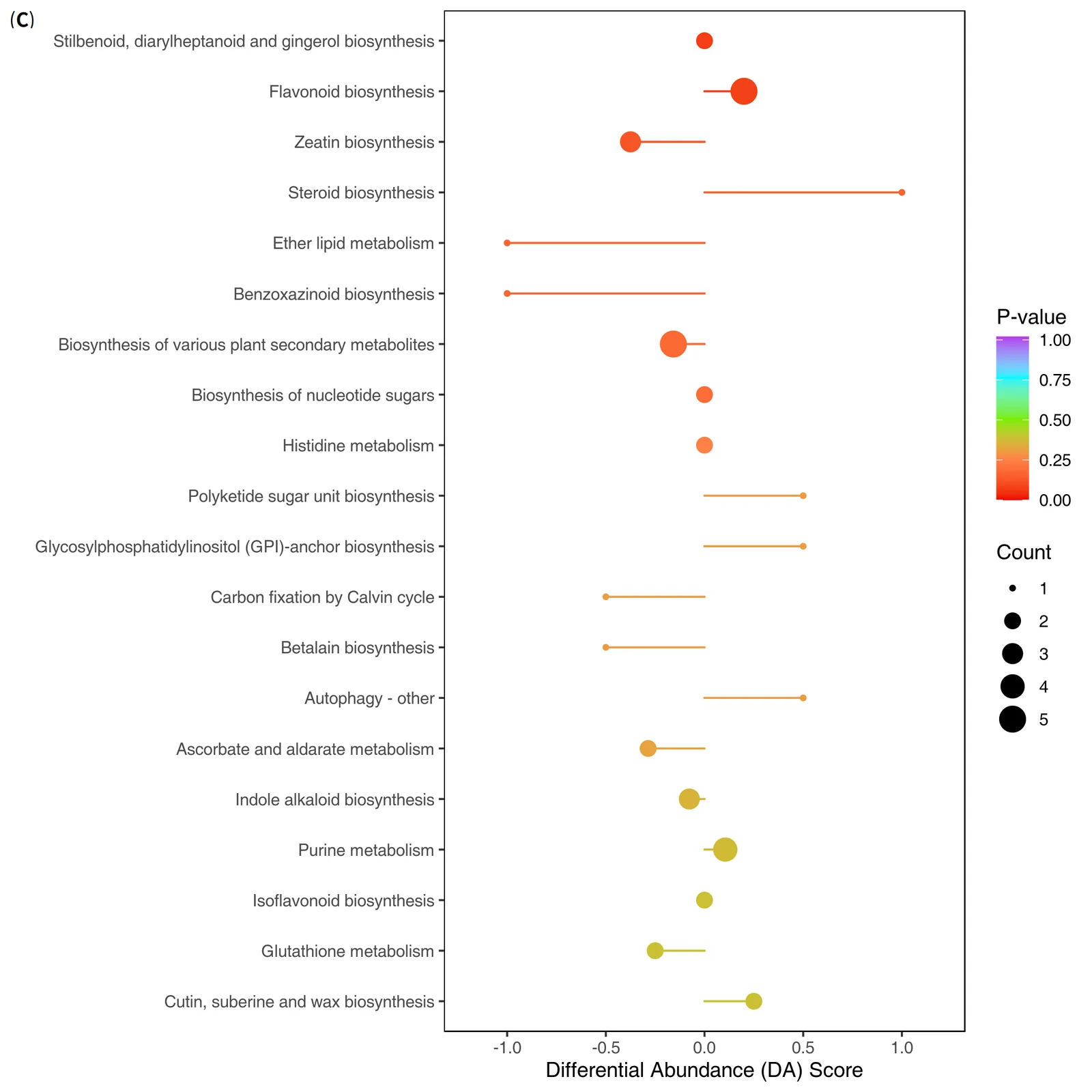
Differential abundance (DA) score of metabolic pathways among different comparison groups. (**A**) GB vs. JD; (**B**) DB vs. JD; (**C**) XJ vs. JD.

KEGG pathway enrichment analysis was performed on differential metabolites from the three comparison groups, and overall pathway trends were evaluated using DA scores (Figure 4). In the GB vs. JD group, the carotenoid biosynthesis pathway showed the greatest down-regulation, whereas the biosynthesis of secondary metabolites pathway exhibited no clear directional change. In the DB vs. JD group, the number of enriched pathways was relatively small and enrichment levels were low, with only a few pathways detected—including biosynthesis of secondary metabolites and phenylalanine/tyrosine/tryptophan biosynthesis—indicating that pathway-level metabolic differences between DB and JD were less pronounced than those between GB and JD, consistent with the PCA results. In contrast, the XJ vs. JD group showed the most significant pathway responses. The flavonoid biosynthesis and stilbenoid, diarylheptanoid, and gingerol biosynthesis pathways—both directly related to phenolic antioxidant activity—exhibited clear up-regulation trends, whereas the isoflavonoid biosynthesis and glutathione metabolism pathways were down-regulated.

Carotenoid and phenolic compound biosynthesis share common precursors such as phenylalanine, which suggests a competitive relationship in carbon metabolic flux [6,22,27]. The down-regulation of the carotenoid biosynthesis pathway in JD, together with the up-regulation of phenolic acids and flavonoids and the widespread down-regulation of lipids, suggests that carbon flux may shift from carotenoid and lipid synthesis toward phenolic secondary metabolism [22]. The DA score of the flavonoid biosynthesis pathway was close to 1.0, indicating that the vast majority of differential metabolites in this pathway were up-regulated in JD [27]. The isoflavonoid biosynthesis pathway showed a down-regulation trend, suggesting selective allocation of phenolic metabolic flux in JD—prioritizing flavonoid synthesis through the main pathway rather than channeling into the isoflavonoid branch [26]. The glutathione metabolism pathway was down-regulated in the XJ vs. JD group, further suggesting that JD’s antioxidant strategy relies more on accumulation of secondary metabolites such as phenolic acids and flavonoids than on small-molecule antioxidants like glutathione [7,8]. Among the three comparison groups, the pathway response in XJ vs. JD was the most intense and specifically targeted activation of the flavonoid biosynthesis pathway. The differences between GB and JD were characterized by global secondary metabolic activation and inhibition of carotenoid synthesis, whereas pathway-level differences between DB and JD were the smallest, suggesting that JD’s metabolic reprogramming pattern exhibits different levels of response depending on the genetic background [6,10]. This carbon reallocation strategy implies that carbon skeletons from primary metabolism (lipid synthesis) are preferentially channeled to support the biosynthesis of phenolic compounds, thereby enhancing the antioxidant reserves and functional quality of the seeds.

### 3.6. Antioxidant Activity and Correlation Analysis of Different Faba Bean Varieties

**Figure 5:**
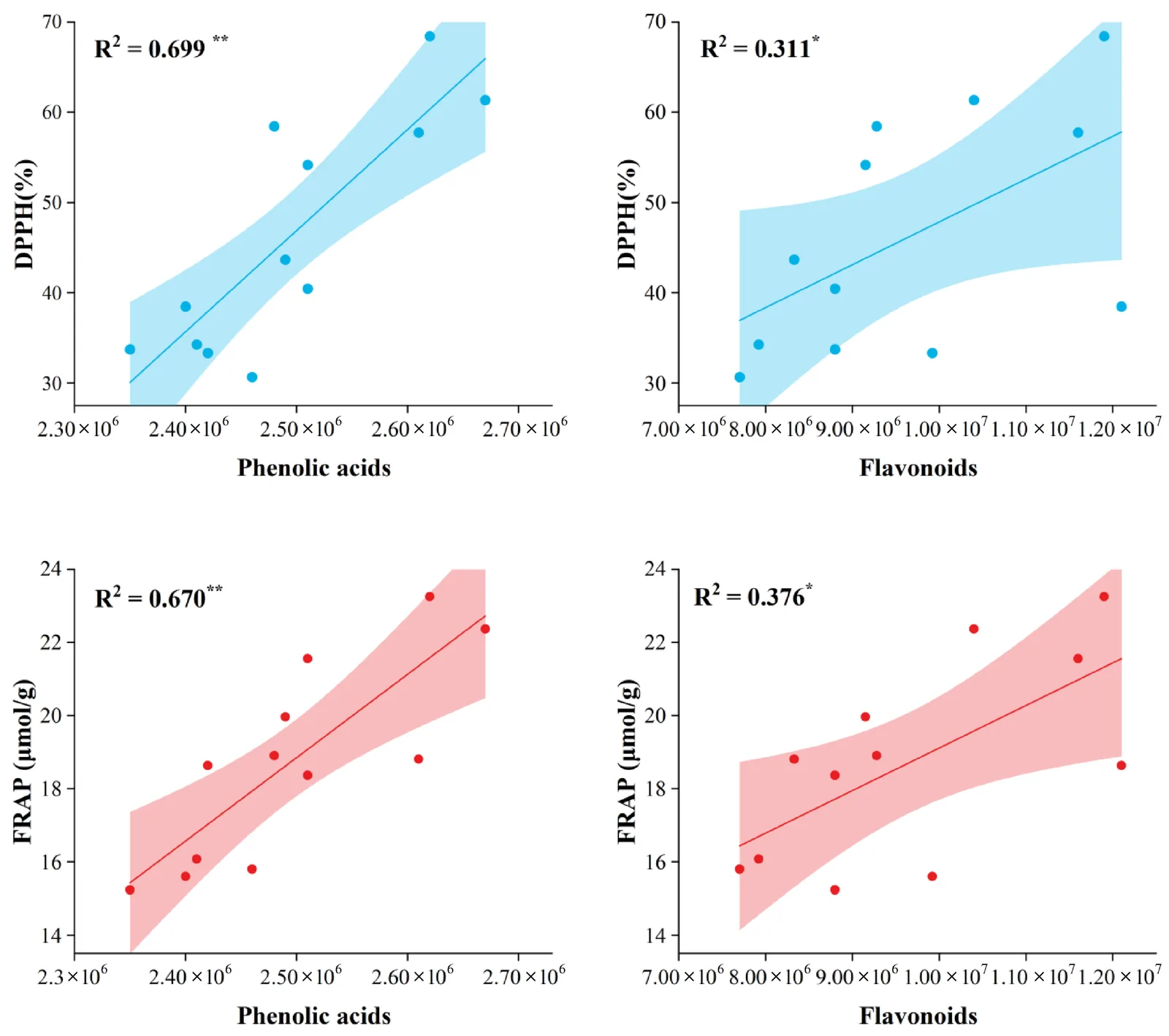
Correlation between phenolic acids, flavonoids, and antioxidant activities. *, ** indicate significant correlations at *p* < 0.05 and *p* < 0.01, respectively.

To validate the antioxidant activity of the up-regulated phenolic acids and flavonoids identified in Section 3.3, we performed DPPH and FRAP assays (Table 3). The DPPH radical scavenging rates and FRAP values of JD and XJ were significantly higher than those of GB and DB (*p* < 0.05), with JD ranking highest in both assays (Figure 5). Correlation analysis revealed that the correlation coefficients between phenolic acid content and DPPH/FRAP values were all above 0.6 (*p* < 0.01), whereas those between flavonoids and antioxidant activity, though also significant (*p* < 0.05), were markedly lower, indicating that phenolic acids and flavonoids jointly contribute to the antioxidant activity of faba bean, with phenolic acids showing stronger correlations and flavonoids providing additional synergistic effects.

To further examine whether this metabolite–phenotype association extends beyond phenolic compounds and antioxidant activity, we performed correlation analysis between eight major metabolite classes and key phenotypic traits, including seed color parameters, nutritional quality (protein, soluble sugar, starch, and total dietary fiber), and antioxidant activities (DPPH and FRAP). The results revealed widespread correlations. Seed color parameters showed stronger correlations with metabolite composition than nutritional indicators. Except for organic acids and derivatives, L* was generally negatively correlated with metabolite classes, while a* and b* were positively correlated, with significant correlations observed for lipids, flavonoids, and terpenoids (*p* < 0.05), indicating that darker seed color is associated with greater metabolite abundance. Nutritional indicators showed relatively weak overall correlations, though some individual correlations reached significance (*p* < 0.05). DPPH and FRAP were positively correlated with all eight metabolite classes, with significant correlations also observed for lipids, amino acids, terpenoids, and alkaloids (*p* < 0.05), in addition to flavonoids and phenolic acids.

Siah et al. [11] previously confirmed that dark-colored faba bean varieties have significantly higher total phenolic content and DPPH scavenging activity than light-colored ones. However, in the present study, XJ exhibited similar phenolic acid content and antioxidant activity to JD, yet its seed color was significantly lighter, suggesting that total phenolic acid content alone does not determine seed color depth. Zanotto et al. [12] reported that phenolic composition differences among faba bean genotypes are partly attributable to variations in polymeric phenolics; however, these compounds were not quantified in the present study. Based on our metabolomic data, several flavonoids (e.g., licochalcone B) were significantly more abundant in JD than in XJ, suggesting that the color difference between JD and XJ may be primarily associated with differential accumulation of flavonoids rather than total phenolic acid content. In addition, JD had significantly higher soluble sugar content than XJ. Soluble sugars can interact with phenolic compounds through hydrogen bonding, affecting their solubility and antioxidant activity, and thereby exerting additional effects on processing properties and flavor [45].

From a practical application perspective, JD—as a newly bred variety—is characterized by darker seed color, high soluble sugar content, and the strongest antioxidant activity, making it suitable for whole flour production, baked functional foods, or natural antioxidant ingredients. XJ, a traditional variety, is distinguished by high protein and high dietary fiber content, with antioxidant activity close to that of JD, making it more suitable as a nutritionally enhanced processing ingredient. GB and DB, as landraces, exhibit stable basic components and relatively low antioxidant activity, and can serve as conventional processing materials [9,17]. Varieties with different genetic backgrounds show distinct patterns in antioxidant accumulation strategies and processing suitability. This multi-trait-based classification provides a clear basis for selecting raw materials for differentiated processing and utilization of faba bean. Collectively, these results confirm that the antioxidant superiority of JD is not merely a function of increased phenolic content, but rather reflects a systematic metabolic configuration—coordinated up-regulation of phenolic pathways, down-regulation of lipid biosynthesis, and enhanced soluble sugar accumulation—that jointly underpins its functional advantage.

### 3.7. Limitations

This study has several limitations. (1) Metabolite annotation was based on putative identification, and targeted absolute quantification using authentic standards and MRM was not performed. (2) Only four varieties were included, and the limited sample size means the conclusions cannot be generalized to the entire faba bean germplasm. (3) Formal statistical correlation analysis between metabolomic and phenotypic data was not conducted, and conventional biochemical quantification of total phenolics and total flavonoids was not performed. Anthocyanins and polymeric phenolics were also not measured; therefore, the interpretation of seed color and antioxidant activity requires further validation through targeted metabolomics and biochemical assays. (4) The antioxidant extraction conditions (solid–liquid ratio, temperature, time, and extraction cycles) were not systematically optimized and could be further refined in future studies. Despite these limitations, the core finding of this study—the consistency between variety-specific metabolomic configurations and quality phenotypes—retains exploratory value and provides directions for future confirmatory research.

## 4. Conclusions

This study systematically evaluated the nutritional quality, antioxidant activity, and metabolomic profiles of four faba bean varieties and confirmed the hypothesis that inter-varietal quality differences are systematically governed by variety-specific metabolomic configurations. JD exhibited the strongest antioxidant activity, which was associated with coordinated up-regulation of phenolic acids and flavonoids, activation of the flavonoid biosynthesis pathway, and suppression of carotenoid biosynthesis. XJ, by contrast, was distinguished by superior crude protein and total dietary fiber contents, representing a complementary quality type. Through TCMSP-based screening, ten JD-specific bioactive compounds, including gallic acid and licochalcone B, were identified as candidate markers for functional quality. These findings provide metabolite-level insights into the chemical basis of quality differentiation in faba bean and establish a metabolomic framework for marker-assisted breeding programs targeting enhanced antioxidant capacity and optimized nutritional profiles. The variety-specific metabolic signatures identified here also offer a rational basis for the targeted utilization of faba bean germplasm in functional food and feed applications.

## Figures and Tables

**Table 1:** Color and nutritional quality characteristics of different faba bean varieties.

Index	JD	GB	DB	XJ
L*	84.45 ± 1.07 b	88.12 ± 2.06 ab	89.64 ± 1.69 a	86.01 ± 0.90 ab
a*	2.04 ± 0.07 a	1.46 ± 0.10 c	1.49 ± 0.13c	1.71 ± 0.12 b
b*	24.60 ± 0.57a	20.94 ± 0.63 b	18.75 ± 0.78 b	22.69 ± 0.31 ab
Protein (%)	30.06 ± 1.53 ab	28.86 ± 1.26 b	29.54 ± 1.23 b	31.78 ± 0.99 a
Soluble sugar (%)	10.38 ± 0.37 a	10.41 ± 0.15 a	9.95 ± 0.56 ab	9.17 ± 0.42 b
Starch (%)	28.91 ± 1.21 a	31.61 ± 1.56 a	30.11 ± 0.58 a	29.08 ± 1.33 a
Total dietary fiber (%)	19.50 ± 0.95 ab	18.36 ± 1.02 b	17.91 ± 1.31 b	21.53 ± 0.78 a

Different lowercase letters in the same row indicate significant differences (*p* < 0.05).

**Table 2:** JD-specific functional metabolites.

No.	Metabolite Name	Category	Functional Activity
1	Gallic acid	Phenolic acids	Classic antioxidant (strongly correlated with FRAP); anti-inflammatory; antibacterial
2	Caffeic acid	Phenolic acids	Antioxidant; anti-cardiovascular disease; a key compound commonly identified in multiple differential metabolite studies
3	Protocatechuic acid	Phenolic acids	Strong antioxidant; one of the main phenolic acids in faba bean seed coats
4	Licochalcone B	Flavonoids	Potent antioxidant (DPPH scavenging); anti-inflammatory (inhibition of NF-κB/COX-2); antitumor
5	Mangiferin	Flavonoids	Antioxidant; antidiabetic; neuroprotective
6	7-Hydroxyflavone	Flavonoids	Antioxidant; anti-inflammatory; estrogenic activity
7	Soyasaponin I	Triterpenoid saponins	Anti-inflammatory (regulating Th1/Th2 balance); immunomodulatory; hypolipidemic
8	Ginsenoside Rh4	Triterpenoid saponins	Antitumor (induces apoptosis); antioxidant; anti-inflammatory
9	Dihydrokaempferol	Flavonoids	Antioxidant (inhibits lipid peroxidation); anti-inflammatory
10	Heptadecanoic acid	Lipids	Antioxidant; PPARδ agonist; anti-inflammatory; mitochondrial repair

**Table 3:** Comparison of antioxidant properties among different faba bean varieties.

Index	Phenolic Acids	Flavonoids	DPPH (%)	FRAP (μmol/g)
JD	2.59 × 10^6^ ± 9.58 × 10^4^ a	1.06 × 10^7^ ± 9.58 × 10^4^ a	62.74 ± 5.13 a	21.51 ± 2.30 a
GB	2.39 × 10^6^ ± 3.27 × 10^4^ b	1.03 × 10^7^ ± 9.58 × 10^4^ a	35.16 ± 2.86 b	16.49 ± 1.87 b
DB	2.42 × 10^6^ ± 4.90 × 10^4^ b	8.14 × 10^6^ ± 9.58 × 10^4^ b	35.12 ± 4.95 b	16.75 ± 1.41 b
XJ	2.54 × 10^6^ ± 6.45 × 10^4^ a	9.70 × 10^6^ ± 9.58 × 10^4^ a	51.86 ± 7.32 a	20.11 ± 1.38 a

Different lowercase letters in the same column indicate significant differences (*p* < 0.05). Phenolic acids and flavonoids are presented as relative contents based on untargeted metabolomics data.

## Data Availability

The original contributions presented in this study are included in the article/Supplementary Material. Further inquiries can be directed to the corresponding author.

## Supplementary Materials

The following supporting information can be downloaded at: https://www.mdpi.com/article/10.3390/plants15182848/s1. Table S1. Information of the four faba bean varieties; Table S2. Contents of vicine and convicine in four faba bean varieties; Table S3. Detailed compound names of the four major categories within the common differential metabolites across the three comparison groups; Figure S1. Total ion chromatograms of the pooled quality control sample; Figure S2. PLS-DA score plot of metabolic profiles among different faba bean varieties; Figure S3. Correlation analysis of major metabolite classes with phenotypic traits in faba bean varieties.
