# Supplementary material for: Variety-Specific Metabolomic Configurations Underlie Differences in Seed Color, Nutritional Quality, and Antioxidant Activity of Faba Bean (Vicia faba L.)
Source: Plants (Basel). 2026 Sep 17;15(18):2848. doi: 10.3390/plants15182848 (PMC13610933; doi:10.3390/plants15182848)
Supplement: Supplementary file 1 [file plants-15-02848-s001.zip › plants-4557947-supplementary.pdf]

Table S1. Information of the four faba bean varieties

Variety code	Origin (Province)	Geographic region	Seed coat color	Variety background	Maturity
JD	Shanxi	North China	Dark brown	breeding line from Shanxi	Late-maturing
GB	Gansu	Northwest China	Light green	Landrace from Gansu	Medium-maturing
XJ	Xinjiang	Northwest China	Light yellow	Introduced variety from Xinjiang	Mid-late maturing
DB	Yunnan	Southwest China	Green	Landrace from Yunnan	Early-maturing

Table S2. Contents of vicine and convicine in four faba bean varieties

Variety	Vicine	Convicine
JD	6.98E+05 b	1.80E+05 b
GB	1.14E+06 a	1.22E+05 c
DB	9.09E+05 a	9.25E+04 c
XJ	9.73E+05 a	2.29E+05 a

Note: Data are presented as relative contents (peak areas) determined by UPLC-MS/MS analysis. Different lowercase letters in the same column indicate significant differences ($p < 0.05$).

Table S3. Detailed compound names of the four major categories within the common differential metabolites across the three comparison groups

Category	Numbers	Metabolite Names	RT (min)	measured m/z
Phenolic acids	7	Gallic acid	1.999	169.014
		Caffeic acid	2.458	341.088
		Protocatechuic acid	2.845	153.019
		3,4-Dimethylbenzoic acid	3.020	195.066
		Phenyl beta-D-glucopyranoside	3.632	533.188
		Trimidox	5.972	223.010
		Cannabidivarin	2.638	287.207
Flavonoids	5	Licochalcone B	2.322	251.069
		Mangiferin	1.481	421.075
		7-Hydroxyflavone	1.207	280.090
		Glepidotin C	5.106	297.153
		4',5,7-Trihydroxy 3,3',6,8-tetramethoxyflavone	1.028	391.109
Organic acids	6	Methylmalonate	4.112	137.024
		Butanoic acid	2.746	556.126
		Cyclamic acid	3.831	224.065
		3-(4-Hydroxy-3-methoxyphenyl)-2-propenoic acid	3.394	193.051
		5-Oxo-4,5-dihydro-2-furylacetic acid	0.657	141.017
		Tetrahydrofolic acid	1.726	446.184
Lipids	7	Heptadecanoic acid	4.360	363.310
		Choline Alfoscerate	0.797	292.072
		22-Hydroxydocosanoic acid	4.674	355.322
		13-Hydroxydocosanoic acid	5.454	393.276
		Gingerglycolipid A	4.647	735.383
		1,2-Dimyristoyl-sn-glycerol	4.478	589.358
		1-Hexadecanoyl-2-(9Z-octadecenoyl)-sn-glycero-3-phosphocholine	4.237	782.568

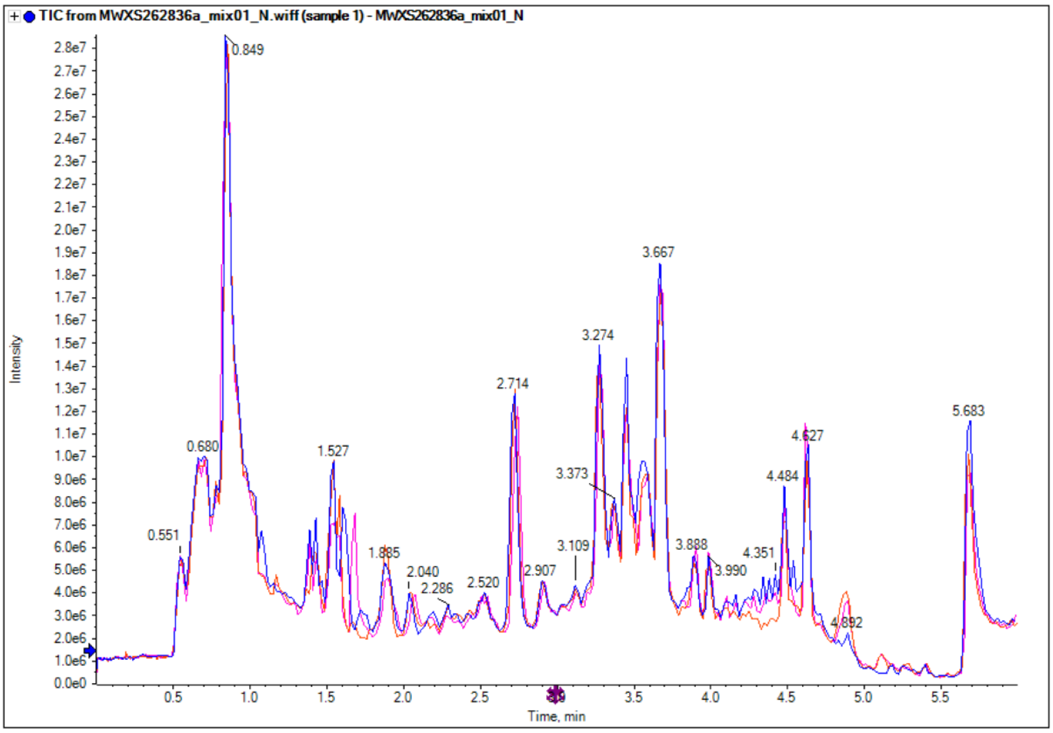

Figure S1. Total ion chromatograms of the pooled quality control sample.

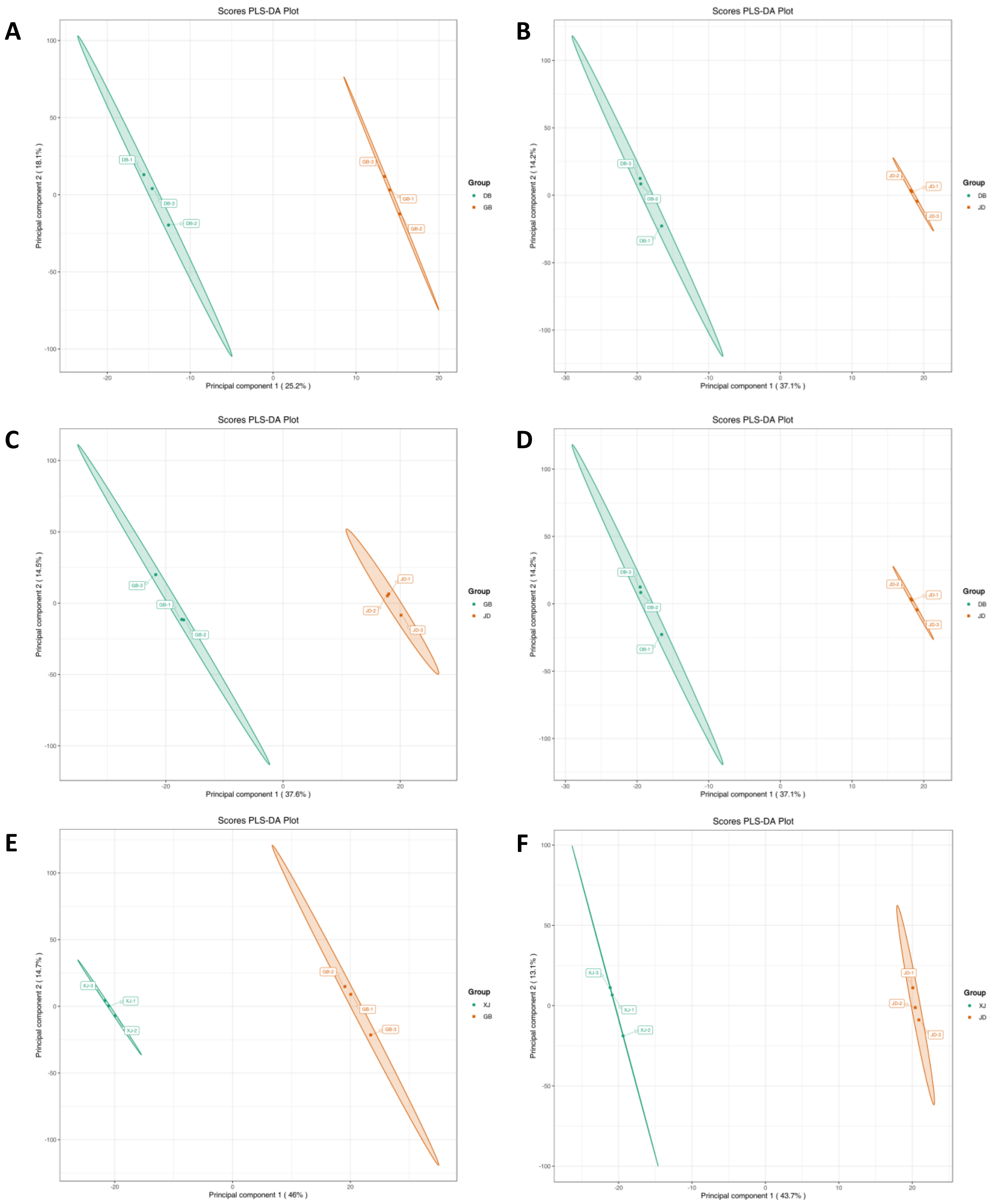

Figure S2. PLS-DA score plot of metabolic profiles among different faba bean varieties. (A) DB vs GB; (B) DB vs JD; (C) GB vs JD; (A) XJ vs DB; (B) XJ vs GB; (C) XJ vs JD.

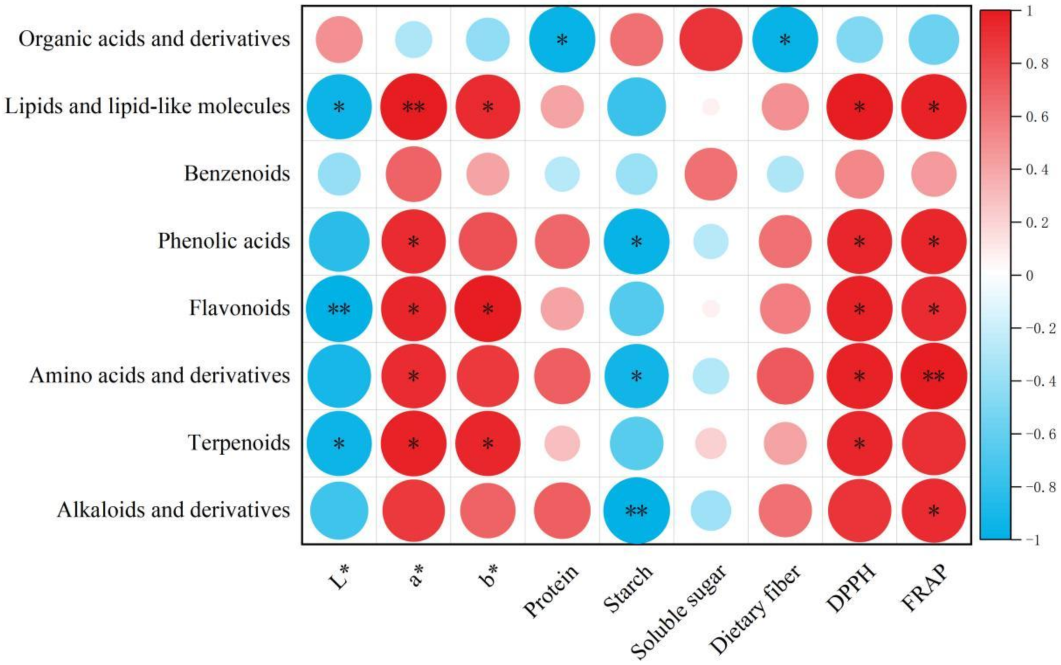

Figure S3. Correlation analysis of major metabolite classes with phenotypic traits in faba bean varieties. * indicates significant correlation at the 0.05 level; ** indicates significant correlation at the 0.01 level.
